# Minimizing Area-Specific Resistance of Electrochemical Hydrogen Compressor under Various Operating Conditions Using Unsteady 3D Single-Channel Model

**DOI:** 10.3390/membranes13060555

**Published:** 2023-05-26

**Authors:** Myungkeun Gong, Changhyun Jin, Youngseung Na

**Affiliations:** Department of Mechanical and Information Engineering, University of Seoul, Seoul 02504, Republic of Korea; pkwy2000@uos.ac.kr (M.G.);

**Keywords:** membrane water content, area-specific resistance, electrochemical hydrogen compressor, single channel

## Abstract

Extensive research has been conducted over the past few decades on carbon-free hydrogen energy. Hydrogen, being an abundant energy source, requires high-pressure compression for storage and transportation due to its low volumetric density. Mechanical and electrochemical compression are two common methods used to compress hydrogen under high pressure. Mechanical compressors can potentially cause contamination due to the lubricating oil when compressing hydrogen, whereas electrochemical hydrogen compressors (EHCs) can produce high-purity, high-pressure hydrogen without any moving parts. A study was conducted using a 3D single-channel EHC model focusing on the water content and area-specific resistance of the membrane under various temperature, relative humidity, and gas diffusion layer (GDL) porosity conditions. Numerical analysis demonstrated that the higher the operating temperature, the higher the water content in the membrane. This is because the saturation vapor pressure increases with higher temperatures. When dry hydrogen is supplied to a sufficiently humidified membrane, the actual water vapor pressure decreases, leading to an increase in the membrane’s area-specific resistance. Furthermore, with a low GDL porosity, the viscous resistance increases, hindering the smooth supply of humidified hydrogen to the membrane. Through a transient analysis of an EHC, favorable operating conditions for rapidly hydrating membranes were identified.

## 1. Introduction

Air pollution and global warming are accelerating as global population growth increases the demand for fossil fuels [1,2]. Inevitably, countries globally are entering a carbon-neutral era. To keep the global average temperature below 2 °C [3] above pre-industrial levels, as stated in the 2015 Paris Agreement [4], many studies over recent decades have focused on new carbon-free energy applications in machinery, power generation, and transportation.

Among the renewable energy sources, hydrogen, which does not release carbon, is the most abundant element in the universe [5] and is a key solution for reducing motor vehicle emissions [6,7]. It can be stored both as a liquid and as a gas, greatly increasing intermittent renewable energy use [8,9]. High-purity hydrogen (>99.97%) is also required for effective operation in mobility applications [10,11]. To use hydrogen in conjunction with renewable energy sources, it is necessary to purify and compress it [12].

**Figure 1:**
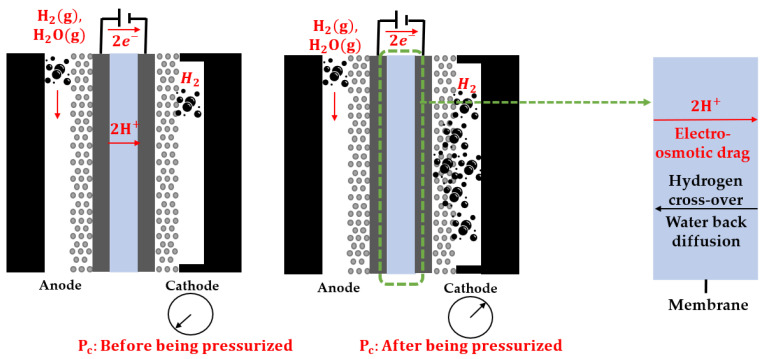
Schematic of an electrochemical hydrogen compressor process.

Physical methods of compressing hydrogen include the use of compressed gas and liquid hydrogen storage [13]. Liquid hydrogen storage can achieve high volumetric densities; however, it is preferred only for special applications because of cost and storage design issues [14]. Therefore, compressed gas storage is the most commonly used storage method [15]. Mechanical and non-mechanical compression methods are used to compress gaseous hydrogen [16] such as hydrogen-embrittled mechanical compressors [17]. In addition, mechanical compressors have many moving parts and tend to leak hydrogen through seals. Hence, electrochemical hydrogen compressors (EHCs) can be used as an alternative [13]. EHCs operate as shown in Figure 1. According to Equation (1), hydrogen is supplied to the anode and oxidized in the catalyst layer. Subsequently, protons move to the cathode through a proton exchange membrane (PEM), and electrons move through an external circuit. According to Equation (2), the electrons and protons are combined and reduced to hydrogen at the cathode. This compression is performed under high pressure at the cathode to improve the specific volumetric energy density of hydrogen. The high-pressure hydrogen compressed at the cathode crosses over to the low-pressure anode, and back diffusion of water occurs due to the concentration difference.

(1) $$\mathrm{Anode}:\;H_{2}\to 2H^{+}+2e^{-}$$

(2) $$\mathrm{Cathode}:\;2H^{+}+2e^{-}\to H_{2}$$

The use of an EHC has various advantages, such as the ability to produce high-purity hydrogen by allowing only proton transmission with a proton exchange membrane. In addition, because there are no moving parts, no lubrication is required, and no noise or minor breakdowns occur. The only limitation is that the energy is wasted because of the back diffusion [17]. In addition, the mechanical strength of the EHC is a major concern when operating at high pressures [18]. To reduce the waste of unused hydrogen from the PEM fuel cell, it can be reused for the EHC [19].

Suermann et al. reported that EHCs have high efficiency (>60%) and low energy consumption in low-pressure applications that do not exceed 10 MPa [20]. Sheffield et al. reported that the density of compressed hydrogen gas is lower than that of liquefied hydrogen, but the energy required for compression is one third that of liquefied hydrogen [14]. Grigoriev et al. developed an EHC capable of compressing hydrogen to 13 MPa at a flow rate of $0.01\;{Nm}^{3}/h$ [21]. Using an EHC, the fuel cell energy was able to attain hydrogen pressures of 88.25 MPa in a single-stage operation while maintaining a hydrogen recovery efficiency exceeding 98% [22]. Giner Inc. reported that an EHC was capable of providing pressures from 87.5 MPa to 140 MPa in a single-stage mode [22]. Although many studies have been conducted in terms of the efficiency and pressurization, the membrane water management problems have rarely been addressed. Ion conduction in proton exchange membranes requires water to facilitate channeling across the entire surface. If water is not evenly distributed throughout the entire membrane, it can impede proton conduction, leading to a decrease in performance. Therefore, effective water management is critical in EHC systems.

To overcome the water management challenges, Yang et al. developed a water-free membrane for EHCs, fuel cells, and electrolyzers. The ionic conductivity of the membrane was 0.01 S/cm at 140 °C, and the proton transport number was 0.17 to 0.2 [23]. They only analyzed the effect of temperature and ignored the effect of the gas diffusion layer (GDL) porosity and relative humidity. Sdanghi et al. studied the effect of the current density supplied to the system, the relative humidity of the inlet hydrogen, and the membrane thickness on the EHC performance. When humidified hydrogen with 90% relative humidity was supplied to the EHC using Nafion 117, its relative humidity decreased to 55% along the gas channel on the anode side, and the current density distribution decreased from 0.75 $A/{cm}^{2}$ to 0.65 $A/{cm}^{2}$. Their study only considered the relative humidity and membrane thickness and not the effect of the temperature and GDL porosity together [24]. In an EHC, hydrogen must be supplied with sufficient hydration [25]. Zavala et al. designed a sulfonated poly (ether-ether ketone, SPEEK) membrane with an increased proton conductivity from 0.014 to 0.027 S/cm and a high water retention capacity [26]. Giner ELX Inc. designed a water management membrane (WAMM) to improve the current density of an EHC from 1 to 1.4 A/$cm^{2}$ [27]. Hao et al. conducted a study using an EHC model with an internal humidifier and found that the main resistance of the EHC was due to the resistance of the electrolyte membrane. When the operating temperature of the cell was increased, the diffusivity of the water improved, and the membrane resistance decreased [28]. Ströbel et al. investigated the effect of the PEM thickness by utilizing several membranes and found that the thicker the PEM, the lower the hydrogen crossover, but the higher the membrane resistance [18]. The main overpotential in an EHC is the ohmic overpotential, which is caused by the conduction of protons through the membrane [29,30]. Therefore, the membrane must be sufficiently saturated with water to achieve high ionic conductivity.

Water management is a key factor in EHC performance because of the dependence of the membrane conductivity on the water content [22]. Previous studies have primarily focused on the development of new membranes. Only a few studies have performed area-specific resistance analysis of EHCs depending on the temperature, relative humidity, and GDL porosity. In addition, there is little research on the relationship between the time taken for the membrane to be hydrated and the performance depending on hydration.

In view of this, this study was conducted to determine the optimal EHC operating conditions that can quickly hydrate the membrane through unsteady-state simulation. This study aimed at investigating the relationship between membrane hydration and EHC performance over time.

## 2. Three-Dimensional Single-Channel Modeling

**Figure 2:**
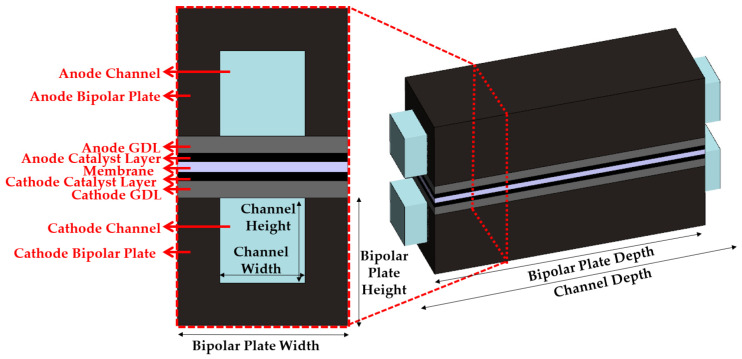
Three-dimensional single-channel computational domain.

A numerical analysis was performed to observe the area-specific resistance of an EHC. Figure 2 shows the EHC single-channel model using SOLIDWORKS 2020. A 3D single channel consists of bipolar plates, gas channels, GDLs, CLs, and membranes. Table 1 lists the geometric parameters of the single-channel EHC model.

## 3. Mathematical Model

### 3.1. Governing Equations

A three-dimensional model was developed to simulate the electrochemical reactions of the EHC. To observe the change in the water content of the membrane over time, a simulation was conducted under the unsteady state. It was assumed that hydrogen is an ideal gas and that the flow is laminar. In addition, the effect of gravity was ignored [31]. STAR-CCM+ 2021.3.1 (16.06.010) was used for computational fluid dynamics (CFD) with the governing equations of the EHC, including the mass, momentum, energy, species, and charge conservation equations.

The conservation of mass equation implies that the sum of all the masses entering and leaving a unit volume in a given time is equal. This can be expressed as follows [32]:

(3) $$\frac{\partial }{\partial t}\left(\varepsilon \rho \right)+\nabla \left(\varepsilon \rho U\right)=0$$

where ε is introduced to describe the GDL region with porosity. The first term is the rate of mass change per unit volume, and the second term is the net rate of mass change per unit volume due to convection. Similar to the mass conservation equation, the momentum conservation equation can be expressed as [32]

(4) $$\frac{\partial }{\partial t}\left(\varepsilon \rho U\right)+\nabla \left(\varepsilon \rho UU\right)=-\varepsilon \nabla p+\nabla \left(\varepsilon \mu \nabla U\right)+\frac{\varepsilon^{2}\mu U}{K}$$

The first term on the left-hand side represents the rate of momentum change per unit volume, and the second term represents the convection term. The first term on the right-hand side represents the net rate of momentum change per unit volume due to pressure, and the second term represents viscous friction. The third term represents Darcy’s law, which quantifies the viscous force exerted on the fluid in a porous structure. The energy conservation equation used to describe thermal equilibrium is as follows [32]:

(5) $$\frac{\partial }{\partial t}\left(\varepsilon \rho h\right)+\nabla \left(\varepsilon \rho Uh\right)=\nabla k^{eff}\nabla T+\varepsilon \frac{\partial p}{\partial t}-j\eta +\frac{ii}{\sigma }+{\dot{s}}_{h}$$

The first term on the right-hand side represents the energy change rate due to heat conduction, and the second term represents the energy change rate obtained from the change in volume due to pressure. The third term represents the heat generated by charge transfer, and the fourth term represents the heat generated by resistance loss. The fifth term represents the entropy loss due to the electrochemical reaction.

The conservation of mass and momentum equations describe the motion of the fluid mixture. The species conservation equation describes the production and consumption of individual species in a fluid mixture and can be expressed as follows [32]:

(6) $$\frac{\partial }{\partial t}\left(\varepsilon \rho X_{i}\right)+\nabla \left(\varepsilon \rho UX_{i}\right)=\nabla (\rho D_{i}^{eff}\nabla X_{i})+{\dot{s}}_{i}$$

The first term on the right-hand side represents the net rate of species mass change per unit volume via diffusion. The second term represents the production and consumption of the chemical species. Assuming that the ion concentration remains unchanged across the membrane, charge conservation can be expressed as [31]

(7) $$\nabla \left(\sigma_{e}^{eff}\nabla \varphi_{e}\right)=j$$

Both the anode and cathode reactions are defined through the Butler–Volmer equation for the electric current density. The Butler–Volmer equation is as follows [33]:

(8) $$j=j_{0}\left\{\exp\left(\alpha_{a}^{eff}\frac{F}{RT}\eta \right)-exp(-\alpha_{c}^{eff}\frac{F}{RT}\eta )\right\}$$

where $\alpha_{a}^{eff}$ and $\alpha_{c}^{eff}$ represent the effective anode and cathode transfer coefficients, respectively. $\alpha_{a}^{eff}$ and $\alpha_{c}^{eff}$ are expressed as follows [33]:

(9) $$\alpha^{eff}=\alpha \cdot n_{e}$$

### 3.2. Boundary Conditions and Numerical Method

The simulation was conducted in galvanostatic mode. Boundary conditions were applied to the single-channel EHC model to observe the membrane water content. Humidified hydrogen was supplied to the anode, and the velocity inlet conditions were applied. Pressure was applied at the anode outlet. The inlet and outlet of the cathode were pressurized by setting the wall boundary conditions. The electrical conductivity, porous viscous resistance, and intrinsic permeability of the GDL were calculated as follows [33]:

(10) $$\sigma_{GDL}=\varepsilon^{1.5}\times 10^{5}$$

(11) $$P_{v}=\frac{\varepsilon^{2}\mu U}{K}$$

(12) $$K=\frac{d_{0}^{2}\varepsilon^{3}}{16k_{K}(1-{\varepsilon )}^{2}}$$

where $k_{K}$ is the Kozeny constant, which was 4.26 in this study. In the membrane, protons drag and move one or more water molecules. This is called electro-osmotic drag. Back diffusion occurs when water accumulates at the cathode and diffuses backward. Electro-osmotic drag and back diffusion can be expressed as follows [32]:

(13) $$J_{H_{2}O}=2n_{drag}^{SAT}\frac{j}{n_{e}F}\frac{\lambda }{22}-\frac{\rho_{MEM}}{M_{MEM}}D_{\lambda }\frac{d\lambda }{dy}$$

where $D_{\lambda }$ is the water diffusivity obtained through experiments, which can be expressed as follows [34]:

(14) $$D_{\lambda }=4.1\times 10^{-10}{\left(\frac{\lambda }{25}\right)}^{0.15}\left[1+tanh\left(\frac{\lambda -2.5}{1.4}\right)\right]$$

The water content can be expressed using the water activity as follows [32]:

(15) $$\lambda =\left\{\begin{array}{c} 0.043+17.81a_{w}-39.85a_{w}^{2}+36.0a_{w}^{3},for\;0<a_{w}\leq 1\\ 14+1.4\left(a_{w}-1\right),for\;1<a_{w}\leq 3 \end{array}\right.$$

The activity of water is calculated as the ratio of the actual water vapor pressure to the saturation water vapor pressure and can be expressed as follows [32]:

(16) $$a_{w}=\frac{p_{w}}{p_{SAT}}$$

The ionic conductivity can be calculated as a function of the water content, and the temperature can be expressed as follows [33]:

(17) $$\sigma_{MEM}=(0.5193\lambda -0.326)e^{1268(\frac{1}{303}-\frac{1}{T})}$$

As the cathode is pressurized, hydrogen crossover occurs because of the pressure difference of the anode, which can be expressed as follows [35]:

(18) $${\dot{n}}_{x}=\left(-2.6492+0.018\left(T-273.15\right)+0.0036RH+0.5992\left(\frac{P_{C}-P_{A}}{100,000}\right)+\frac{10.84}{\ln\left(t_{MEM}\right)}\right)\times 10^{-9}$$

In this study, the catalyst layer was treated as a solid phase, and the electrochemical reactions were modeled to occur at the interface between the porous GDL and the solid catalyst layer. The physical properties and operating conditions of the EHC model are listed in Table 2.

To select an appropriate mesh and time step, the Courant–Friedrichs–Lewy (CFL) number was obtained using Equation (19):

(19) $$CFL=\frac{u\cdot ∆t}{∆x}\leq 1$$

where u is the velocity magnitude, ∆t is the time step, and ∆x is the interval length of the computational mesh. The CFL number of the coarse grid of (43,198 cells) and time step of (0.001 s) was 1, and that of the coarse grid of (43,198 cells) and time step of (0.004 s) was 4. In addition, the CFL number of the fine grid of (130,070 cells) and time step of (0.004 s) was 12.12. In this study, an implicit solver was used. In this case, the accuracy remains unaffected even if the CFL number is larger than one, and Poinsot et al. reported that the accuracy is the best when the CFL number is four [36,37].

**Figure 3:**
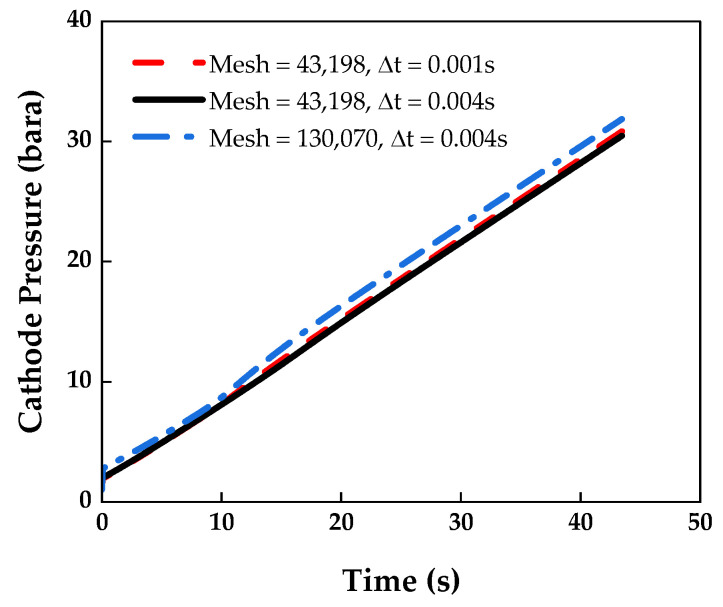
Mesh and time step independence tests at a current of 0.0315 A.

Figure 3 shows the results of the mesh and time step independence tests for the cathode pressure over time. The coarse grid of (43,198 cells) and time step of (0.001 s) differed by approximately 1% from the coarse grid of (43,198 cells) and time step of (0.004 s). In addition, there was a 5% difference in the coarse grid of (43,198 cells) and time step of (0.004 s) from the fine grid of (130,070 cells) and time step of (0.004 s). Therefore, the simulation was conducted by selecting the course grid of (43,198 cells) and time step of (0.004 s) to shorten the calculation time.

## 4. Results and Discussion

### 4.1. Validation of Model

The membrane water content was analyzed depending on the operating temperature, relative humidity, and GDL porosity. A temperature of 333.15 K, a relative humidity of 100%, a polymer electrolyte membrane thickness of 125 μm, and a GDL porosity of 0.4 were selected as the reference conditions of the model. A polymer electrolyte membrane thickness of 125 μm is widely used commercially [38]. As 125 μm membranes exhibit low specific energy consumption at low current densities, a 125 μm membrane was applied in this simulation [2].

**Figure 4:**
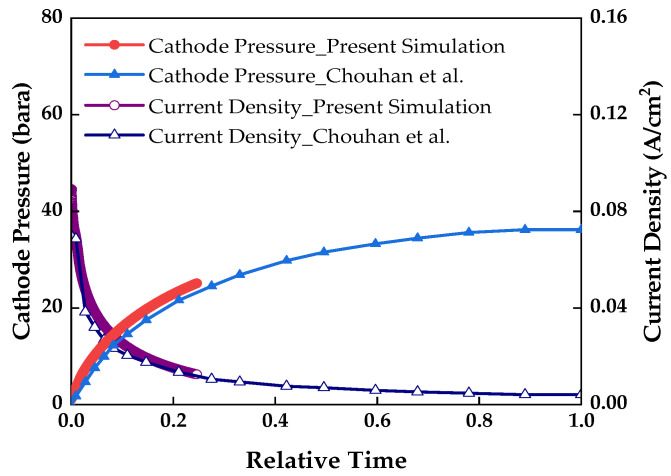
Comparison of the cathode pressure and current density over relative time through simulation with the results of Chouhan et al. [39], at a voltage of 0.05 V.

To verify the reliability of the 3D unsteady-state model, we compared the results of the present simulation with the experimental results of Chouhan et al. [39]. Figure 4 shows the results of the cathode pressure and current density over time when the voltage was 0.05 V; the results are based on the reference model. Due to the difference in the cathode volume, the simulation results showed a faster convergence time for the cathode pressure and current density than that in the results of Chouhan et al. [39]. Therefore, the results are expressed in relative time, which was obtained by dividing the time taken for the cathode pressure and current density to converge on the x-axis. When compared with the results of Chouhan et al., the cathode pressure matched well with a deviation 1.4%, and the validity of the simulation model was verified [39].

### 4.2. Operating Temperature Effects

**Figure 5:**
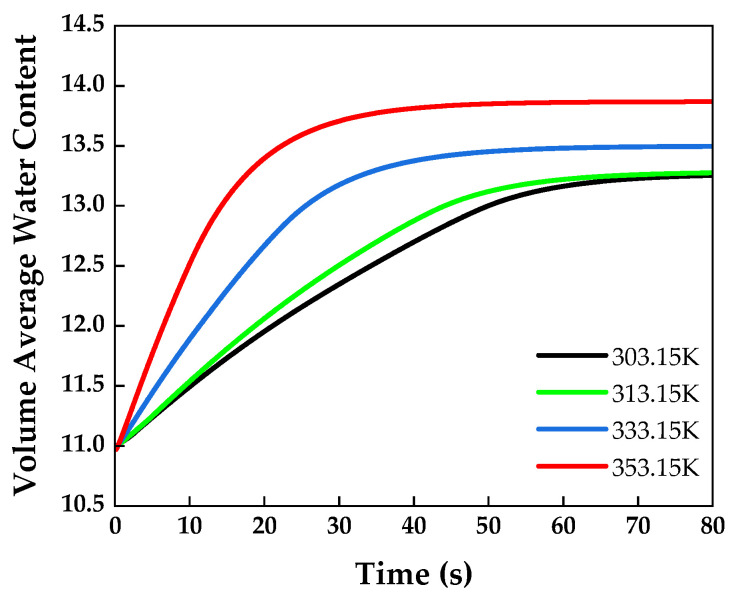
Volume-averaged water content over time measured in the membrane at various temperatures.

To analyze the membrane water content accumulation according to the temperature, numerical analysis was performed under the following operating conditions: a relative humidity of 100%, a polymer electrolyte membrane thickness of 125 μm, and a GDL porosity of 0.4. Figure 5 shows the volume-averaged water content across the membrane over time, measured at various temperatures, which increased rapidly as the operating temperature increased. This is because the amount of saturated water vapor supplied increases with the temperature. Therefore, the volume-averaged water content was the highest at 353.15 K.

**Figure 6:**
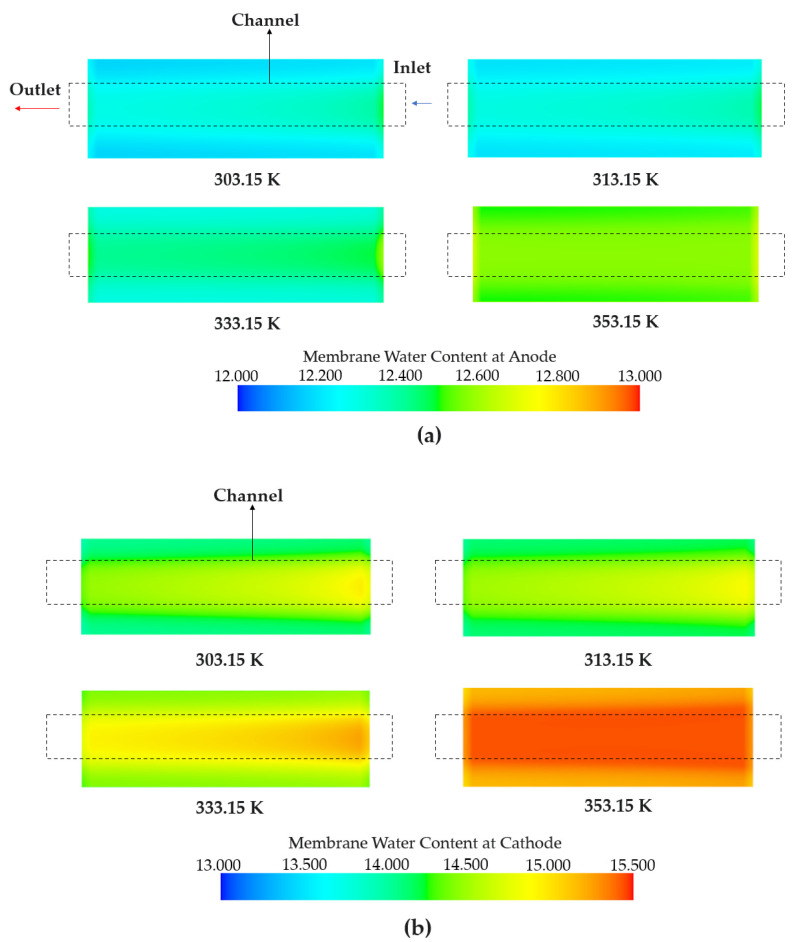
Water content depending on the various operating temperatures observed at 80 s. (**a**) Anode water content. (**b**) Cathode water content.

The water content is a nonlinear function of the water activity [40]. The water activity is expressed as a function of the water saturation partial pressure and water partial pressure calculated from the temperature [41]. As the water content is a nonlinear function of the temperature, 303.15 K and 313.15 K converged to similar values. As shown in Figure 6, the water content of the anode and cathode had similar values at 303.15 K and 313.15 K. In addition, higher temperatures contribute to a higher water content owing to the increased water vapor supply.

**Figure 7:**
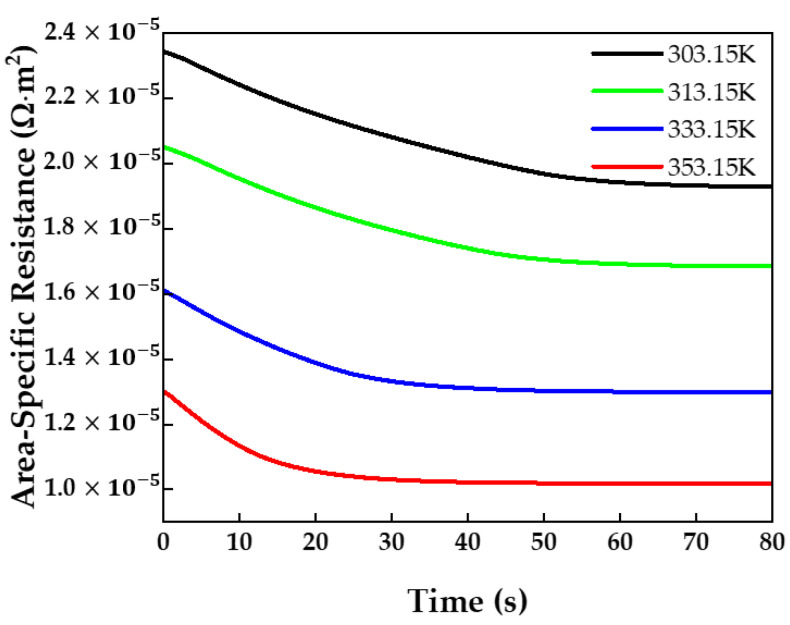
Membrane area-specific resistance over time at different operating temperatures.

As shown in Figure 7, when the time was at 0 s, the area-specific resistance varied depending on the temperature, while the volume-averaged water content showed no dependence on the temperature. As the ionic conductivity includes a temperature term, the area-specific resistance varied largely with the temperature. Thus, a difference of about 47.2% was observed between 303.15 K and 353.15 K in terms of the area-specific resistance. Consequently, as the operating temperature increased, the area-specific resistance decreased.

### 4.3. Relative Humidity Effects

**Figure 8:**
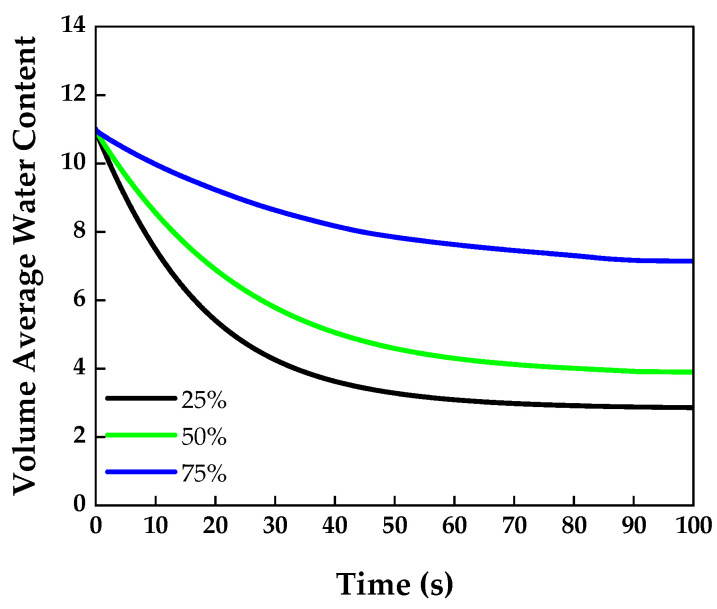
Volume-averaged water content over time measured in the membrane at various relative humidities.

A temperature of 333.15 K, a polymer electrolyte membrane thickness of 125 μm, and a GDL porosity of 0.4 were selected to compare the water content results depending on the relative humidity. Figure 8 shows the water content over time for each relative humidity level. As a low relative humidity leads to a low partial pressure of water vapor, the water content dropped significantly in the case of 25% relative humidity compared to that of the other cases.

The relative humidity results show that the volume-averaged water content decreased with the time, differing from the temperature results in Section 4.2. In this study, the initial volume-averaged water content was set to 11. The reason for this is because Khan S et al. [42] stated that the membrane water content is maintained at 11 under sufficiently humidified conditions. Therefore, when water vapor was supplied at a relative humidity of less than 100%, the water content decreased over time.

**Figure 9:**
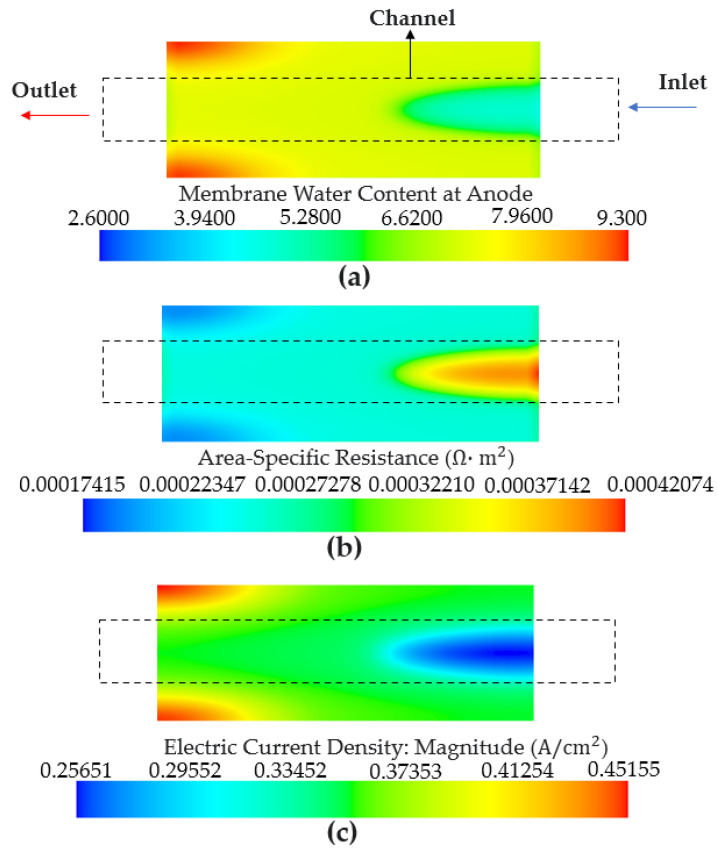
Simulation results under a relative humidity of 75% at the anode observed at 100 s. (**a**) Membrane water content. (**b**) Area-specific resistance. (**c**) Electric current density.

Figure 9a shows the water content of the membrane with a relative humidity of 75%. The inlet region exhibited a drier state compared with the other regions. As mentioned earlier, the membrane was properly hydrated with a volume-averaged water content of 11 in the initial stage. Generally, when dry water vapor is supplied to a membrane that is already adequately hydrated, the inlet region becomes drier compared to the other regions. Due to the poor diffusion of the dry water vapor towards the edge of the outlet region, the water content in that region remained higher compared to the other regions. Hence, the area-specific resistance shown in Figure 9b indicates a high value in the inlet region and a low value at the edge of the outlet region. Additionally, Figure 9c further illustrates that the dried inlet region had a low current density, whereas a high-current-density distribution was observed at the edge of the outlet region.

**Figure 10:**
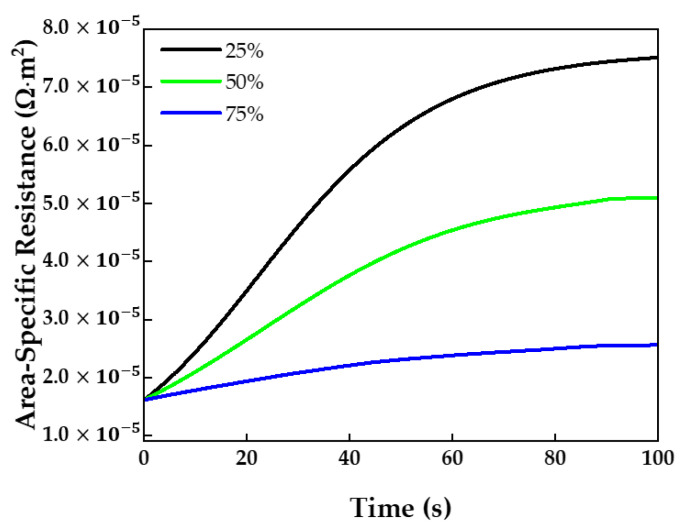
Membrane area-specific resistance over time at different relative humidities.

The higher the relative humidity, the higher the induced partial pressure. As shown in Figure 10, the increase in the area-specific resistance was slower at a high relative humidity than at a lower RH. Therefore, a high relative humidity should be selected to sufficiently hydrate the membrane.

### 4.4. GDL Porosity Effects

**Figure 11:**
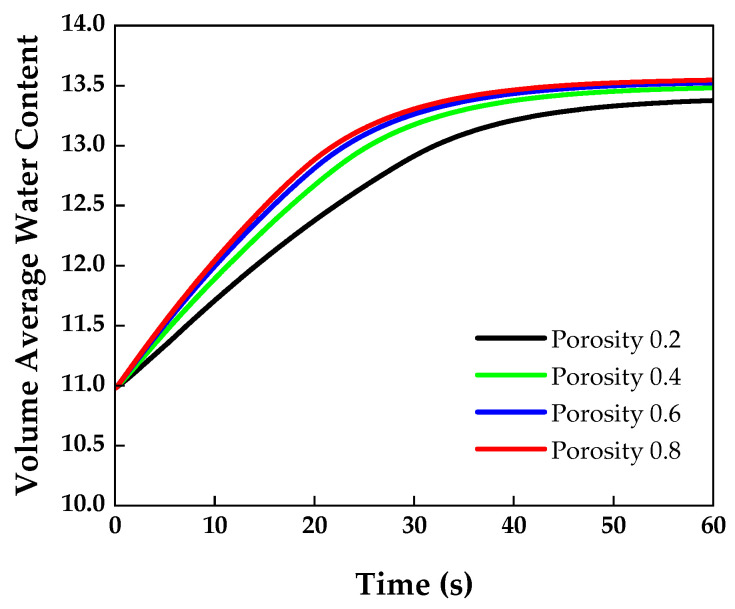
Volume-averaged water content over time measured in the membrane at various GDL porosities.

The GDL undergoes changes in the conduction of heat, electrical, water, and gas transport, depending on the material properties [43]. Figure 11 shows the volume-averaged water content at various GDL porosities.

**Figure 12:**
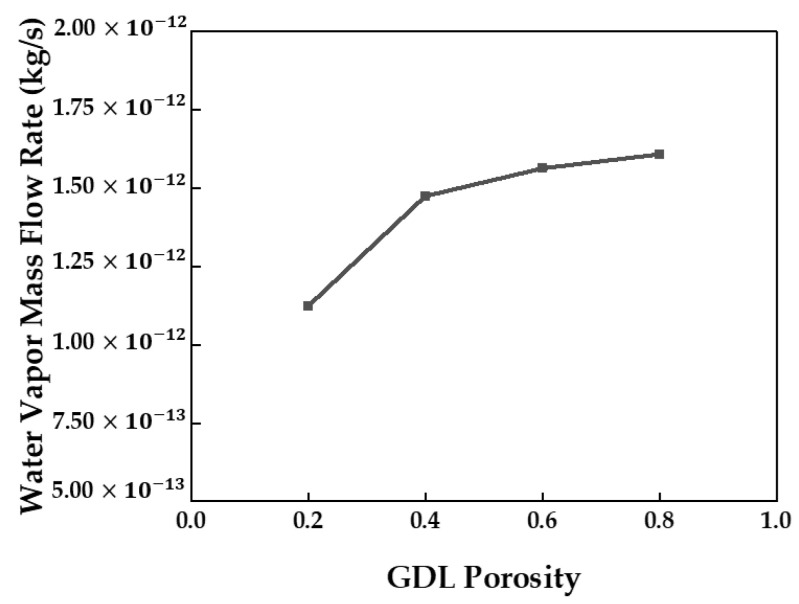
Water vapor mass flow rate depending on the GDL porosity measured at the channel–GDL interface.

**Figure 13:**
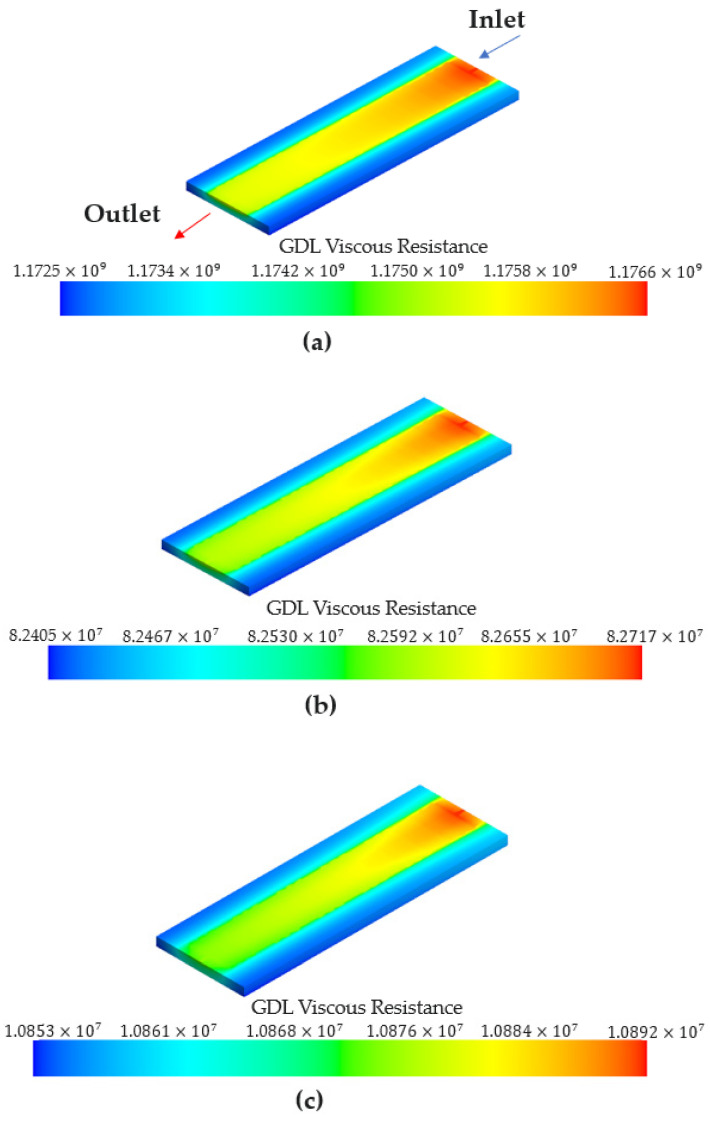

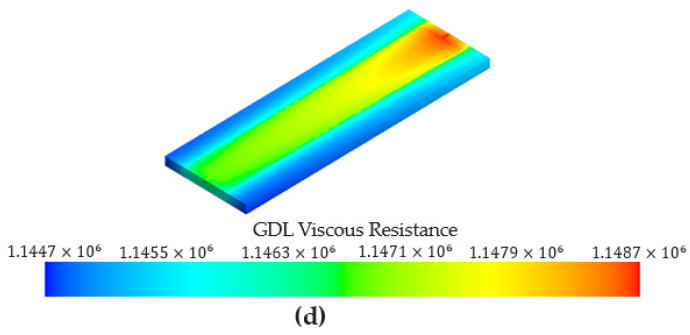
Viscous resistance measured in the GDL depending on the GDL porosity at 60 s. (**a**) Porosity of 0.2. (**b**) Porosity of 0.4. (**c**) Porosity of 0.6. (**d**) Porosity of 0.8.

The lower the GDL porosity, the lower the volume-averaged water content measured in the membrane. When the GDL porosity was low, the amount of water vapor passing through the GDL area decreased. Figure 12 shows the water vapor mass flow rate with respect to the GDL porosity. The water vapor mass flow rates under GDL porosities of 0.4, 0.6, and 0.8 were similar. As shown in Figure 13, the highest viscous resistance was obtained for a GDL porosity of 0.2. Therefore, the lowest water vapor mass flow rate was observed. In addition, as the velocity term of the viscous resistance is included in Equation (11), a higher viscous resistance was obtained at the inlet where a high flow rate was observed. The viscous resistance then gradually decreased towards the outlet.

**Figure 14:**
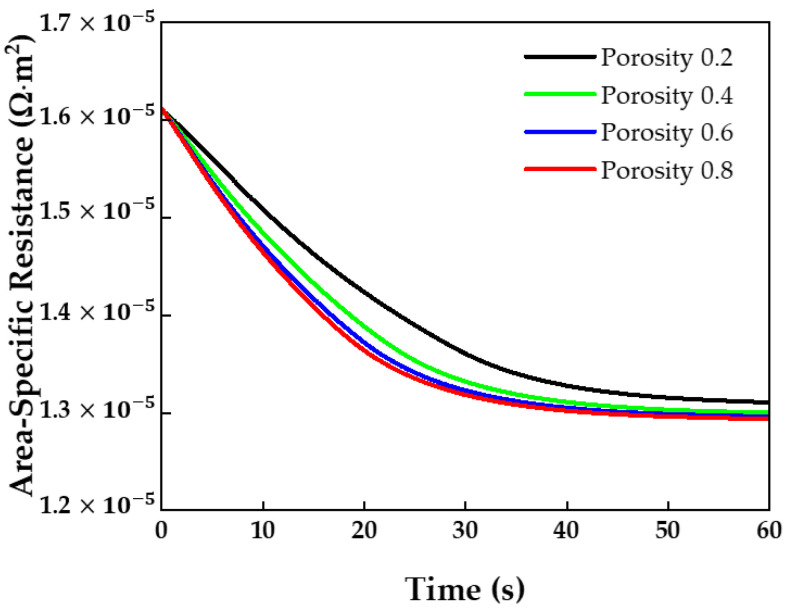
Membrane area-specific resistance over time at different GDL porosities.

Figure 14 shows the results of the measurement of the area-specific resistance over time using various GDL porosities. The higher the GDL porosity, the lower the area-specific resistance. A high GDL porosity can smoothly supply water vapor and hydrogen to the membrane and reduce mass transfer resistance [44]. As the current increases, the mass transfer resistance becomes dominant [45]. Therefore, to reduce the area-specific resistance and mass transfer resistance when operating at a high current, it is optimal to use a GDL porosity of 0.4 to 0.8.

## 5. Conclusions

Simulations were conducted using a 3D single-channel model to determine the optimal conditions for rapidly hydrating an EHC membrane. The operating temperature, relative humidity, and GDL porosity were used as parameters. To evaluate the optimal conditions, the volume-averaged water content and area-specific resistance were used as performance indicators.

According to the simulations, depending on the operating temperature, the volume-averaged water content increased as the temperature increased. Thus, a higher temperature contributes to faster hydration of the membrane. In contrast, the area-specific resistance rapidly decreased with increasing operating temperature. Consequently, when the temperature increased, the membrane was rapidly hydrated, whereas the area-specific resistance rapidly decreased. A simulation based on the relative humidity was conducted in the fully hydrated state of the membrane. The volume-averaged water content rapidly decreased, whereas the area-specific resistance quickly increased when the relative humidity was 25%. When the membrane is sufficiently hydrated to operate an EHC, 100% humidified water vapor must be supplied. If the porosity is low, water vapor cannot be smoothly supplied to the membrane. Accordingly, the volume-averaged water content increased slowly, and the area-specific resistance decreased slowly. The porosity of the GDL must be high to efficiently hydrate the membrane.

Therefore, maintaining a high water content at all times is necessary to reduce the area-specific resistance. In addition, the membrane is rapidly hydrated as the temperature, relative humidity, and GDL porosity increase. No water is produced by the EHC. Water management is therefore critical. The optimal conditions for efficient water management in EHCs were derived in this study. In the future, studies related to the improvement of EHC efficiency through flow-field design will be conducted with artificial intelligence, similar to the study of Pang et al. [46].

## Figures and Tables

**Table 1:** Geometric properties of the EHC.

Parameter	Units	Value
Bipolar Plate Width × Depth × Height	mm	1.5 × 6.0 × 1.5
Channel Width × Depth × Height	mm	1.0 × 7.0 × 1.0
Thickness of GDL ($t_{GDL}$)	mm	0.2
Thickness of Catalyst Layer ($t_{CL}$)	mm	0.01
Thickness of Membrane ($t_{MEM}$)	mm	0.125

**Table 2:** The physical parameters and operating conditions of the EHC.

Parameter	Units	Value
Faraday Constant (F)	C/mol	96,485.332
Gas Constant (R)	J/mol∙K	8.3144
Exchange Current Density $\left(j_{0}\right)$	$A/{cm}^{2}$	1 [31]
Thermal Conductivity of Bipolar Plate $\left(k_{BP}\right)$	W/m∙K	95 [31]
Electrical Conductivity of Bipolar Plate $\left(\sigma_{BP}\right)$	S/cm	1250 [33]
Thermal Conductivity of Gas Diffusion Layer $\left(k_{GDL}\right)$	W/m∙K	24 [33]
Gas Diffusion Layer Porosity (ε)	-	0.2/0.4/0.6/0.8
Thermal Conductivity of Catalyst Layer $\left(k_{CL}\right)$	W/m∙K	2.37 [33]
Electrical Conductivity of Catalyst Layer $\left(\sigma_{CL}\right)$	S/cm	1 [33]
Dry Density of Membrane $\left(\rho_{MEM}\right)$	$kg/m^{3}$	2000 [33]
Equivalent Weight of Membrane ($M_{MEM})$	kg/kmol	1100 [33]
Operating Temperature (T)	K	303.15/313.15/333.15/353.15
Relative Humidity (RH)	%	25/50/75/100
Inlet Velocity	m/s	1
Operating Current	A	0.0315

## List of Symbols

$a_{w}$	Activity of water/-
$d_{o}$	Fiber diameter/μm
$D_{\lambda }$	Water diffusivity/$m^{2}\;s^{-1}$
$D_{i}^{eff}$	Effective diffusivity for species i/$m^{2}\;s^{-1}$
F	Faraday constant/$A\;s\;{mol}^{-1}$
*h*	Fluid enthalpy/$J\;{mol}^{-1}$
j	Current density/$A\;m^{-2}$
K	Intrinsic permeability of the GDL/$m^{2}$
$n_{e}$	Number of electrons in hydrogen/-
${\dot{n}}_{x}$	Hydrogen crossover flux/mol $s^{-1}\;m^{-2}$
$P_{A}$	Anode pressure/bar
$P_{C}$	Cathode pressure/bar
$P_{v}$	Porous viscous resistance of the GDL/$kg\;m^{-2}s^{-2}$
$p_{w}$	Actual water vapor pressure/bar
$p_{SAT}$	Saturation water vapor pressure/bar
R	Ideal gas constant/$J\;{mol}^{-1}\;K^{-1}$
$t_{MEM}$	Thickness of membrane/mm
T	Temperature/K
U	Velocity vector/$m\;s^{-1}$
$X_{i}$	Mass fraction for species *i*/-
**Greek letters**	
α	Transfer coefficient/-
$\alpha_{a}^{eff}$	Effective transfer coefficient of anode
$\alpha_{c}^{eff}$	Effective transfer coefficient of cathode
ε	Porosity/-
η	Overpotential/V
λ	Water content/-
μ	Viscosity coefficient/$kg\;m\;s^{-1}$
ρ	Density/$kg\;m^{-3}$
σ	Ionic conductivity/$S\;{cm}^{-1}$
$\varphi_{e}$	Membrane potential/V
